# Predicting the prognosis in patients with sepsis by an endoplasmic reticulum stress gene signature

**DOI:** 10.18632/aging.205252

**Published:** 2023-11-25

**Authors:** Jian Liu, Hao Wang, Huimin Xiao, Li Ji, Yonghui Yao, Chunshui Cao, Yong Liu, Liang Huang

**Affiliations:** 1 Department of Emergency, First Affiliated Hospital of Nanchang University, Nanchang 330006, China

**Keywords:** sepsis, endoplasmic reticulum stress, signature, prognosis, biomarkers

## Abstract

Background: Prognostic stratification of patients with sepsis is important for the development of individualized treatment strategies. Endoplasmic reticulum stress (ERS) plays a key role in sepsis. This study aimed to identify a set of genes related to ER stress to construct a predictive model for the prognosis of sepsis.

Methods: The transcriptomic and clinical data of 479 sepsis patients were obtained from GSE65682 and divided into a training set (n=288) and a validation set (n=191) at a ratio of 3:2. The external test set was GSE95233 (n=51). LASSO and Cox regression analyses were performed to establish a signature to predict the prognosis of patients with sepsis. Moreover, we developed a nomogram that included the risk signature and clinical features to predict survival probability.

Results: A prognostic signature was constructed with ten endoplasmic reticulum related genes (ADRB2, DHCR7, GABARAPL2, MAOA, MPO, PDZD8, QDPR, SCAP, TFRC, and TLR4) in the training set, which significantly divided patients with sepsis into high- and low-risk groups in terms of survival. This signature was validated using validation and external test sets. A nomogram based on the risk signature was constructed to quantitatively predict the prognosis of patients with sepsis.

Conclusions: We constructed an ERS signature as a novel prognostic marker for predicting survival in sepsis patients, which could be used to develop novel biomarkers for the diagnosis, treatment, and prognosis of sepsis and to provide new ideas and prospects for future clinical research.

## INTRODUCTION

Sepsis is a systemic inflammatory response syndrome caused by a major injury to the body, including burns, shock, severe infection, severe trauma, and surgery; the inflammatory response occurs rapidly and can quickly progress to severe sepsis and septic shock [1, 2]. Globally, there are estimated to be 31.5 million cases of sepsis annually, and in an estimated 17% and 26% of these cases, patients are hospitalized because of sepsis or severe sepsis, respectively [3]. The results of epidemiological studies indicate that more than half of all ICU deaths are caused by sepsis and its complications [4]. The number of deaths caused by sepsis and septic shock is estimated to be 1,400 worldwide each day [5]. Although sepsis, severe sepsis, and septic shock are now better understood and managed, the incidence of sepsis continues to increase [6]. Currently, a great deal of difficulty is faced when treating patients with severe sepsis and septic shock [7]. Hence, there remains an urgent need for better markers of sepsis and further investigation of the molecular mechanisms underlying sepsis.

The endoplasmic reticulum (ER) plays an important role in cellular protein synthesis and processing, as well as maintaining intracellular stability [8, 9]. In ER stress (ERS), as a result of disruption of ER homeostasis protein folding is compromised, which has been found to be associated with serious pathological processes, including hypoxia, starvation, and calcium imbalance [10, 11]. ER stress has also been confirmed in patients with sepsis. Recognizing the role of ER stress in the pathology of sepsis is important [12]. Lu et al. [13] found that inhibition of ER stress inhibits apoptosis and immune dysregulation in sepsis. Qian et al. [14] showed that ER-related proteins are significantly expressed in the liver of rats with sepsis, and inhibition of endoplasmic reticulum stress can reduce liver cell apoptosis. Recently, various diagnostic and prognostic models based on gene expression data have been developed for different diseases and have shown accurate disease risk prediction [15–17]. However, there has been little research on the role of endoplasmic reticulum stress-related genes in predicting the prognosis and diagnosis of sepsis.

To bridge this knowledge gap, we focused on investigating the association between expression profiles of ERS-related genes (ERGs) and clinical outcomes in sepsis patients and constructed and validated ERGs risk score for predicting sepsis patient survival. This approach and tool are expected to improve the ability for predicting the survival of sepsis patients and guide comprehensive sepsis therapeutic strategies. Finally, a prognostic nomogram and visualization model were created using a web-based calculator integrating the signature, and clinical factors were developed in patients with sepsis.

## MATERIALS AND METHODS

### Data sources

We retrospectively analyzed publicly available transcriptome profiling data (blood samples) and related clinical parameters from the GEO database (https://www.ncbi.nlm.nih.gov/geo/). Two public datasets, GSE65682 (n=760, only 479 sepsis patients with complete data for survival status) and GSE95233 (n=51), including data from 811 sepsis patients, were selected. Raw transcriptional expression profiles with clinical data were used as screening criteria, and the mean value was used when more than one probe was for a gene.

The Biological Information Database of Sepsis (BIDOS, http://www.swmubidos.com/), a database of transcriptome profiling data of patients with sepsis, was used to explore the diagnostic and prognostic roles of individual ERGs in sepsis. ERGs were acquired from the GeneCards Database (https://www.genecards.org/) [18], and 1,116 ERGs with a relevance score ≥ 7 were selected.

### Differentially expressed ERGs and functional analysis

Differentially expressed ERGs were screened by comparing healthy control (n=42) and sepsis samples (n=760) and identified using the “limma” package in the R with adjusted P value < 0.05 and |log2-fold change (FC)|>0.5 as the selection criteria. A heatmap and volcano map were drawn using the R packages “pheatmap” and “ggpubr”.

The “org.Hs.eg.db,” “clusterProfiler,” “enrichplot,” and “ggplot2” R packages were used to perform Gene ontology (GO) and KEGG pathway analysis for differentially expressed ERGs. Statistical significance was set at P < 0.05.

### Establishment and verification of the ERG signature

The patients with sepsis in GSE65682 dataset (n=479, patients with complete survival data) were randomized divided into two groups at a 6:4 ratio: the training set (n=288) and validation set (n=191) using “caret” R package with proportionate-stratified random sampling (https://cran.r-project.org/web/packages/caret/index.html). A comparison of the baseline demographics and clinical profiles of the training and validation sets is presented in Table 1. The training set was used to establish the prognostic ERG signature. Univariate Cox regression analysis was conducted to select the survival-related ERGs by the “survival” package. LASSO regression was also used to screen for significant candidate genes to analyze survival profiles by the R package “glmnet”, which can display actively associated variables in higher dimensions. Subsequently, a multivariate Cox regression risk signature was built based on selected genes using LASSO regression to assess the prognosis of sepsis patients by calculating the risk score. A formula was developed using the gene signatures described above.

**Table 1:** Baseline demographics and clinical characteristics of patients in training set and validation set.

**Characteristics**	**Training set (n=288)**	**Validation set (n=191)**	**Total (n=479)**	**P value**
Gender, n (%)				0.13
Male	172 (35.91%)	100 (20.88%)	272 (56.78%)	
Female	116 (24.22%)	91 (19.00%)	207 (43.22%)	
Age, years	60.04±15.40	62.34±13.74	60.95±14.79	0.09
Pneumonia diagnoses, n (%)				0.92
HAP	47 (9.81%)	30 (6.26%)	77 (16.08%)	
CAP	62 (12.94%)	44 (9.19%)	106 (22.13%)	
Unknown	179 (37.37%)	117 (24.43%)	296 (61.80%)	
ICU acquired infection, n (%)				0.61
No	161 (33.61%)	114 (23.80%)	275 (57.41%)	
Yes	27 (5.64%)	19 (3.97%)	46 (9.60%)	
Unknown	100 (20.88%)	58 (12.11%)	158 (32.99%)	
Diabetes, n (%)				0.26
No	178 (37.16%)	123 (25.68%)	301 (62.84%)	
Yes	50 (10.44%)	39 (8.14%)	89 (18.58%)	
Unknown	60 (12.53%)	29 (6.05%)	89 (18.58%)	
Outcome, n (%)				0.12
Dead	61 (12.73%)	53 (11.06%)	114 (23.80%)	
Alive	227 (47.39%)	138 (28.81%)	365 (76.20%)	

HAP, hospital acquired pneumonia; CAP, community acquired pneumonia.

Risk score = mRNA1×coefficient1+ mRNA2×coefficient2+……+ mRNA× coefficient i.

Based on the median risk score, patients with sepsis were separated into low- and high-risk categories. The sensitivity and specificity of the prognostic signature were determined using Kaplan-Meier curve analysis and ROC curves. The predictive accuracy the ERG risk model was assessed by Kaplan-Meier curve and ROC analysis.

The risk score was also calculated using the validation set. Based on the cutoff value from the training cohort, the cases in the validation set were separated into high- or low-risk groups.

### Immune infiltration analysis

To determine difference of immune infiltration between different risk groups, a violin plot was constructed to present the contribution of immune cell infiltration in the high- and low-risk groups using the CIBERSORT algorithm (https://cibersort.stanford.edu/) [19]. The 22 types of immune cells were mapped using a landscape map to show how they differed between high- and low-risk groups.

### Statistical analysis

Our statistical analyses were conducted using R software (version 4.2.1) and GraphPad Prism 8.3.0. Result with P < 0.05 (two-sided) were considered statistically significant.

## RESULTS

### Identification of differentially expressed genes

**Figure 1:**
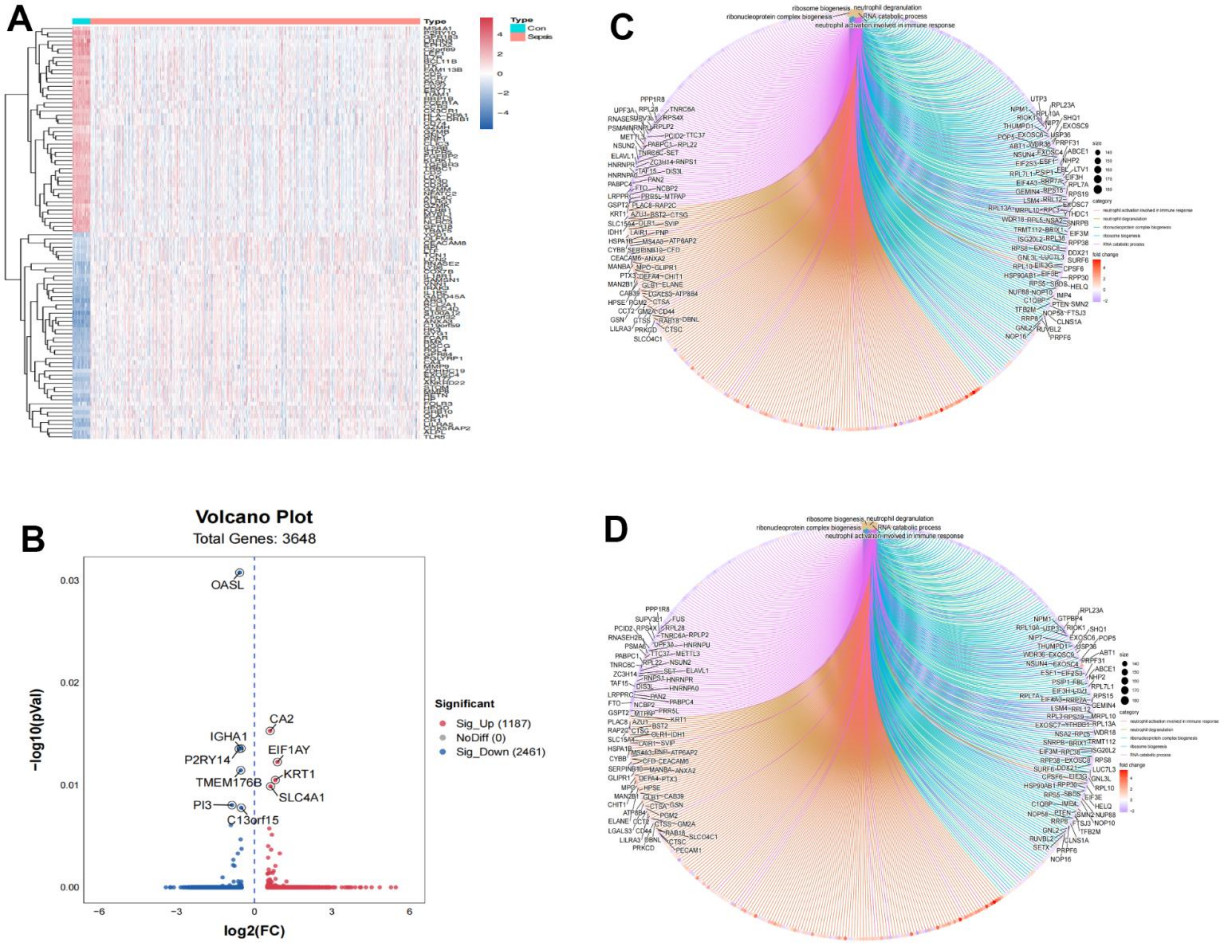
**Identification of differently expressed genes associated with sepsis.** (**A**) Heatmap of top 100 differently expressed genes. (**B**) Volcano plot of 3,648 differently expressed genes. (**C**) GO functional enrichment of differently expressed genes between normal control and sepsis. (**D**) KEGG functional enrichment of differently expressed genes between normal control and sepsis.

To obtain the differentially expressed genes between healthy subjects and sepsis, gene expression of GSE65682 datasets were enrolled as discovery dataset. A total of 3648 genes, including 1187 up-regulated and 2461 down-regulated genes, were screened in GSE65682 dataset between healthy control and sepsis (Supplementary Table 1). Heatmap was used to indicate the top 100 differential genes in sepsis (Figure 1A). The volcano plot was generated for the differential genes (Figure 1B). To explore the function of this differentially expressed genes, the dysregulated genes were then used to perform GO and KEGG pathway enrichment analyses, separately (Figure 1C, 1D).

### Construction of ERG prognostic signature

First, according to the expression levels of the 256 differentially expressed ERGs (Supplementary Figure 1A), duration of follow-up, and survival status acquired from GEO database, we got a total of 64 ERGs related to survival by using univariate analysis (Supplementary Table 2). Then, the 64 survival related ERGs were submitted to LASSO regression by 10-fold cross validation, and 16 ERGs were identified (Supplementary Figure 1B). Finally, the selected 16 ERGs used for multivariate Cox regression analysis and 10 ERGs were included in the risk signature, eventually (Supplementary Figure 1C). Based on the Cox regression, we established an ERGs signature as follows: risk score = (-0.66117 × mRNA value of ADRB2) + (0.23933 × mRNA value of DHCR7) + (-1.08258 × mRNA value of GABARAPL2) + (0.30108 × mRNA value of MAOA) + (0.12385 × mRNA value of MPO) + (0.92966 × mRNA value of PDZD8) + (-0.53967 × mRNA value of QDPR) + (-0.63250 × mRNA value of SCAP) + (0.30857 × mRNA value of TFRC) + (-0.53252 × mRNA value of TLR4).

Moreover, we investigated the correlations between the 10 identified ERGs. The heat map of gene expression correlation is showed in Supplementary Figure 1D.

### Validation of the expression of the identified ERGs

To verify the microarray gene expression data, the 10 genes were selected for validation by BIDOS database by meta-analysis. By pooling the data of all eligible studies, the results revealed that ADRB2 mRNA were significantly decreased for the sepsis cases in comparison with normal cases (Supplementary Figure 2A, SMD=0.67, 95% CI=[0.49,0.95]). By pooling the data of all eligible studies, the results revealed that DHCR7 mRNA were significantly increased for the sepsis cases in comparison with normal cases (Supplementary Figure 2B, SMD=-0.76, 95% CI = [-0.99, -0.52]). By pooling the data of all eligible studies, the results revealed that GABARAPL2 mRNA were significantly increased for the sepsis cases in comparison with normal cases (Supplementary Figure 2C, SMD=-0.74, 95% CI=[-0.98, -0.50]). By pooling the data of all eligible studies, the results revealed that MAOA mRNA were significantly increased for the sepsis cases in comparison with normal cases (Supplementary Figure 2D, SMD=-0.27, 95% CI = [-0.54,0.00]). By pooling the data of all eligible studies, the results revealed that MAOA mRNA were significantly increased for the sepsis cases in comparison with normal cases (Supplementary Figure 2E, SMD=-0.97, 95% CI=[-1.21, -0.73]). By pooling the data of all eligible studies, the results revealed that PDZD8 mRNA were significantly increased for the sepsis cases in comparison with normal cases (Supplementary Figure 2F, SMD=-0.94, 95% CI=[-1.18,-0.70]). By pooling the data of all eligible studies, the results revealed that QDPR mRNA were significantly decreased for the sepsis cases in comparison with normal cases (Supplementary Figure 2G, SMD=0.54, 95% CI=[0.31,0.78]). By pooling the data of all eligible studies, the results revealed that SCAP mRNA were significantly decreased for the sepsis cases in comparison with normal cases (Supplementary Figure 2H, SMD=0.91, 95% CI=[0.68,1.15]). By pooling the data of all eligible studies, the results revealed that TFRC mRNA were significantly increased for the sepsis cases in comparison with normal cases (Supplementary Figure 2I, SMD=-0.50, 95% CI=[-0.72,-0.27]). By pooling the data of all eligible studies, the results revealed that TLR4 mRNA were significantly increased for the sepsis cases in comparison with normal cases (Supplementary Figure 2J, SMD=-0.92, 95% CI=[-1.16,-0.67]).

### ERGs diagnostic value for sepsis

In order to determine the diagnostic value of the 10 selected ERGs in the risk signature, the ROC curve was employed based on GSE65682. Supplementary Figure 3 showed the AUC value of ADRB2 (0.928 [95%CI: 0.896-0.961]), DHCR7 (0.794 [95%CI: 0.750-0.839]), GABARAPL2 (0.898 [95%CI: 0.862-0.934]), MAOA (0.913 [95%CI: 0.876-0.951]), MPO (0.815[95%CI: 0.759-0.871]), PDZD8 (0.893[95%CI: 0.857-0.928]), QDPR (0.956 [95% CI: 0.932-0.981]), SCAP (0.924 [95% CI: 0.900-0.947]), TFRC (0.715 [95%CI: 0.674-0.757]), and TLR4 (0.829 [95%CI: 0.786-0.872]).

### ERGs prognostic value for sepsis

**Figure 2:**
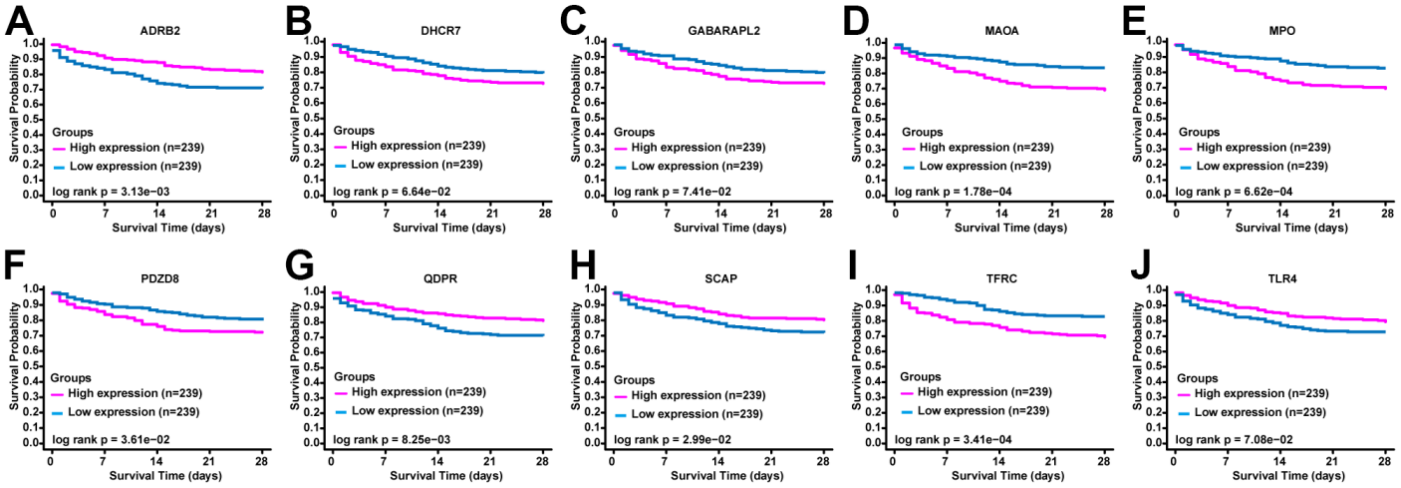
**The prognostic value of ERGs expression in sepsis.** KM survival curves of (**A**) ADRB2, (**B**) DHCR7, (**C**) GABARAPL2, (**D**) MAOA, (**E**) MPO, (**F**) PDZD8, (**G**) QDPR, (**H**) SCAP, (**I**) TFRC, and (**J**) TLR4.

To verify the prognostic value of the selected ERGs, we performed the overall survival curves based on the data from BIDOS database. Survival analyses revealed that sepsis cases with low levels of mRNA of ADRB2, QDPR, SCAP, and TLR4 had worse overall survival (Figure 2). However, sepsis cases with increased levels of mRNA of DHCR7, GABARAPL2, MAOA, MPO, PDZD8, and TFRC had worse overall survival (Figure 2).

### Predictive power of the ERG signature

**Figure 3:**
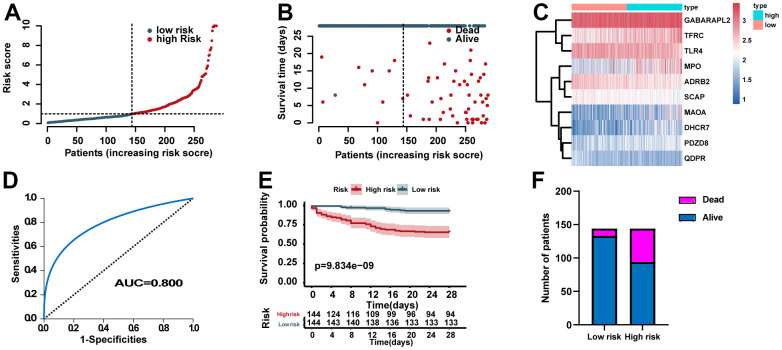
**Assessment of the predictive power of the risk signature in the training set.** (**A**) Distribution of risk scores. (**B**) Survival status of each case. (**C**) Heat map of ERG expression. (**D**) ROC analysis of the survival-related prognostic signature. (**E**) Kaplan–Meier (KM) survival curves for the overall survival of the risk score. (**F**) Comparison of survival rate between two groups.

According to the median of the ERG signature score, the training set was categorized into high- and low- risk groups. The distribution of risk scores, survival status of each case, and the heat map of ERGs expression in the training set are summarized in Figure 3A–3C. We further performed ROC analysis to evaluate the predictive power of the ERG based risk signature and drew ROC curve. The results showed that the AUC of the survival-related ERG signature was 0.800 for the training set (Figure 3D). The KM analysis confirmed that the survival curve for the low-risk cases was higher than that for the high-risk cases (Figure 3E). Figure 3F also showed the proportion of dead cases was increased in the high-risk group.

### Validation of the prognostic role of the risk signature

**Figure 4:**
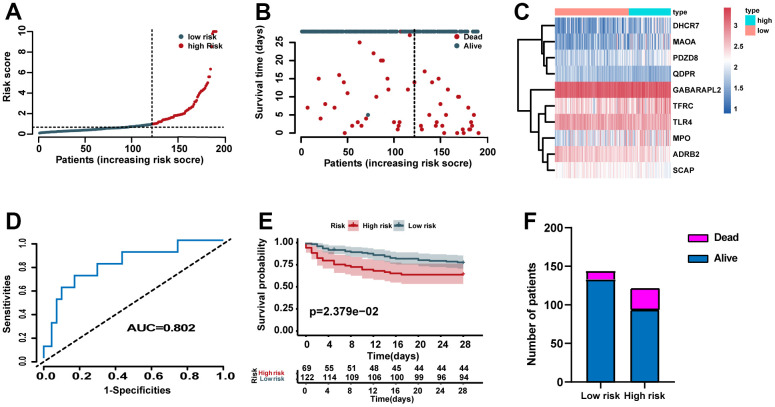
**Validation of the predictive power of the risk signature in the validation set.** (**A**) Distribution of risk scores. (**B**) Survival status of each case. (**C**) Heat map of ERG expression. (**D**) ROC analysis of the survival-related prognostic signature. (**E**) Kaplan–Meier (KM) survival curves for the overall survival of the risk score. (**F**) Comparison of survival rate between two groups.

According to the identified cutoff value in the training set, the cases for validation set was categorized into high- and low- risk groups. The distribution of risk scores, survival status of each case, and the heat map of ERGs expression in the training set are showed in Figure 4A–4C. In addition, we carried out ROC analysis to evaluate the predictive power of the ERG based risk signature and drew ROC curve. The results showed that the AUC of our ERG signature was 0.802 in the validation set (Figure 4D). The KM analysis confirmed that the high-risk cases had a worse prognosis when compared with the low-risk cases (Figure 4E). Figure 4F showed the proportion of dead cases were increased in the high-risk group.

**Figure 5:**
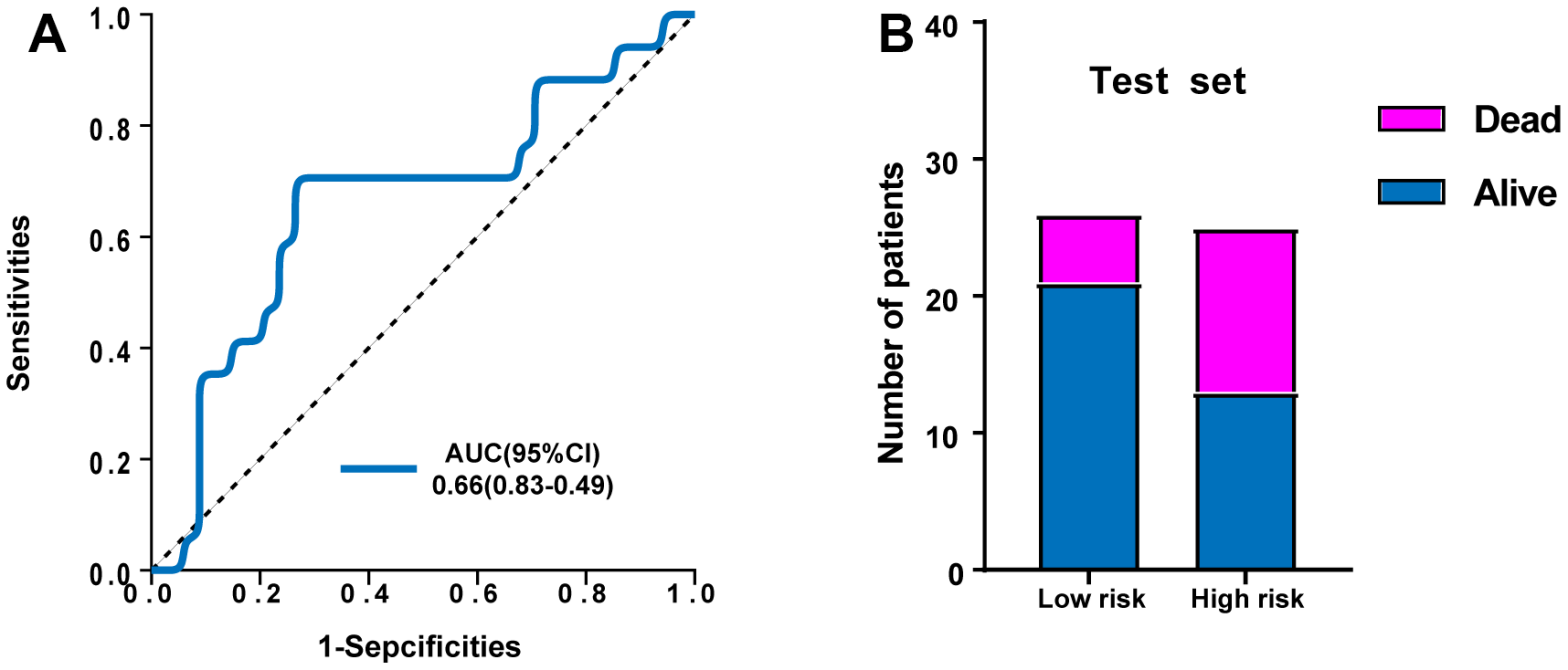
**Validation of the predictive power of the risk signature in the external test set.** (**A**) ROC analysis of the survival-related prognostic signature. (**B**) Comparison of survival rate between two groups.

In addition, the GSE95233 dataset was used as an external dataset. We performed ROC analysis to evaluate the predictive power of the ERG based risk signature and drew ROC curve. The results showed that the AUC of our novel ERG signature was 0.660 for GSE95233 (Figure 5A). Figure 5B also showed the proportion of dead cases was increased in the high-risk group.

### The patterns of immune cell infiltration

**Figure 6:**
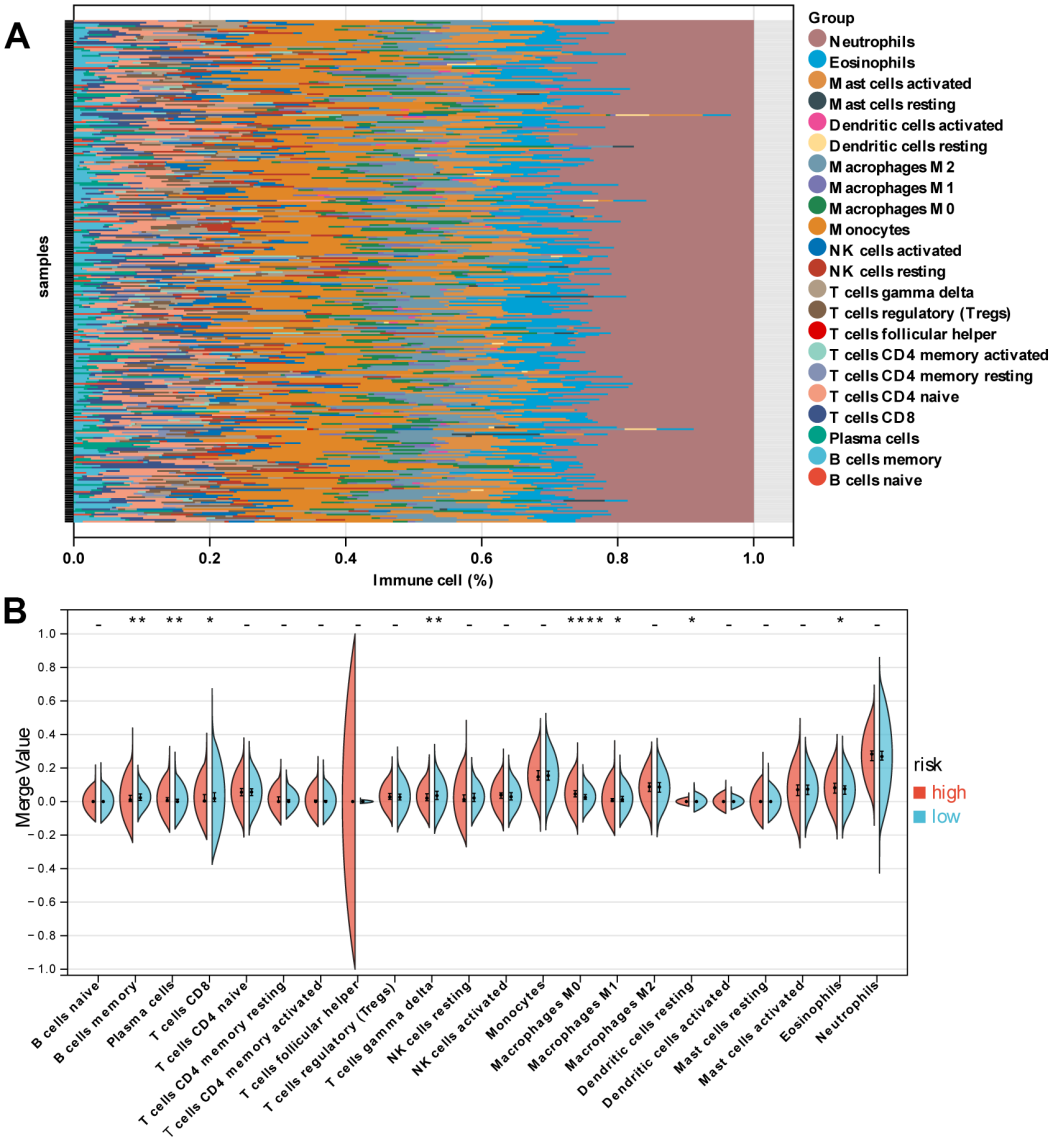
**Immune characteristics of patients in the high- and low-risk groups.** (**A**) Relative proportion of immune cells between high- and low-risk groups. (**B**) Violin plot indicating relative proportions of immune cell expression distribution of sepsis patients stratified by the ten-gene signature into high- and low-risk groups.

Figure 6A revealed the patterns of 22 subpopulations of immune cells’ proportion in blood between sepsis and normal control. Violin plot of absolute CIBERSORT scores revealed that compared to low-risk groups, the high risk exhibited higher infiltration of plasma cells, M0 macrophages, Dendritic cells resting, and Eosinophils, and lower infiltration of B cells memory, T cells CD8, T cells gamma delta, and Macrophages M1 (Figure 6B).

### ERGs signature acts as an independent prognostic predictor

**Figure 7:**
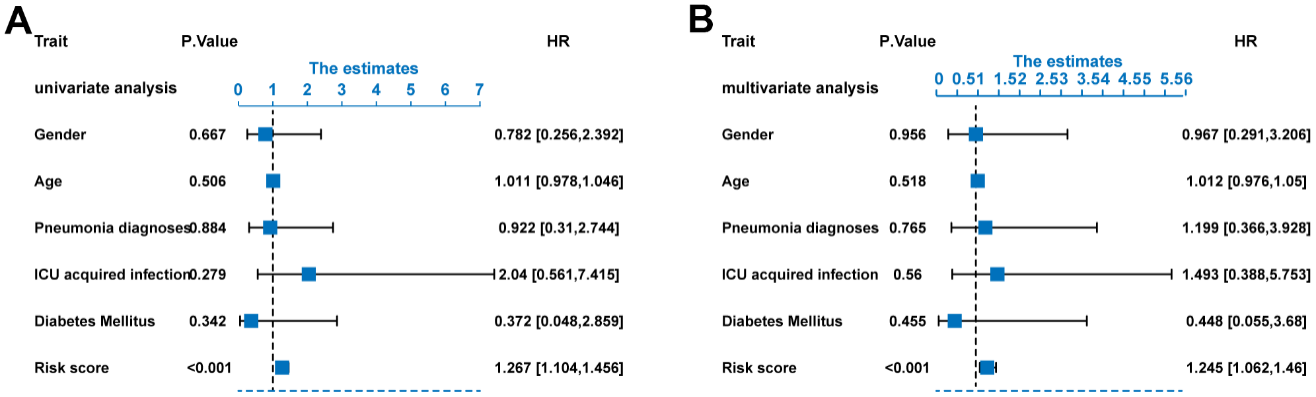
**Risk score as an independent predictor of survival.** (**A**) Univariate Cox regression analysis results. (**B**) Multivariate Cox regression analysis results.

In order to find the independent prognostic factors for sepsis, we performed univariate and multivariate Cox regression analysis in the training set based on the following factors: gender, age, pneumonia, ICU acquired infection, diabetes, and ERG signature score. The results indicated that the ERG signature was an independent of risk factor for outcome of sepsis (Figure 7A, 7B).

### Construction of nomogram

To better predict the survival probability of different time of sepsis patients, we established a nomogram based on the gender, age, pneumonia, and risk signature (Supplementary Figure 4A). With the median risk score as the cut-off value, the training set cases were divided into high risk and low risk. The KM analysis showed that the survival curve of the low-risk group was higher than that of the high-risk group (Supplementary Figure 4B). Supplementary Figure 4C shows the Calibration curves of the nomogram for the probability of survival at 5-, 15-, and 25- days. We performed ROC analysis to assess the predictive power of the nomogram and drew ROC curve. The results revealed that the AUC of 5-, 15- and 25- days survival predictive power was 0.871, 0.862, and 0.833, respectively (Supplementary Figure 4D). It is conceivable that the final combined prognostic model is more efficient than single risk signature.

**Figure 8:**
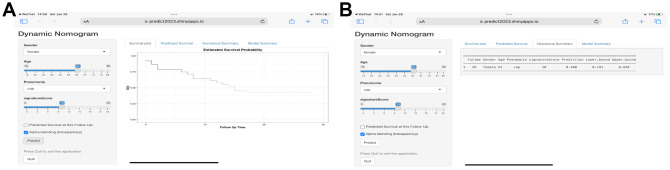
**Web-based calculator for estimating the survival probability.** (**A**) By entering the specifics of the sepsis into the web-based program, the participant’s survival probability can be determined (https://predict2023.shinyapps.io/survival/). (**B**) Numerical summary of the model.

Finally, we have also created an online predict tool for users to explore and visualize the predictive data (Figure 8) (https://predict2023.shinyapps.io/survival/).

## DISCUSSION

Sepsis is the most common causes of death for critically ill patients [20, 21]. Early identification of diagnostic and prognostic biomarkers of sepsis can play a pivotal role in its treatment, and when appropriate treatment is promptly initiated, the mortality rate reduces [22, 23]. Recently, high-throughput sequencing and data processing, which can identify biomarkers for prognosis prediction, have gradually become significant tools in biomedical research [24, 25]. Accordingly, there is now a significant opportunity to develop biomarkers as predictive indicators of sepsis and to use these biomarkers as the foundation for the development of effective therapies.

In the present study, we got the mRNA expression data and survival data from the GEO database to screen the prognostic-related ERGs. Then, LASSO and multivariate Cox regression analyses were carried out based on the selected ERGs. Next, we identified a ten-gene signature (ADRB2, DHCR7, GABARAPL2, MAOA, MPO, PDZD8, QDPR, SCAP, TFRC, and TLR4), rather than a single gene, with prognostic value for cases of sepsis. The stability and efficiency of the prediction model were verified using independent GEO datasets. The results of Cox regression analyses found the ten-ERG risk score was an independent risk predictor for outcome of sepsis. Lastly, we established a nomogram to help us more intuitively predict the 5 -, 15-, and 25-day survival rates. To the best of our knowledge, our work is the first study which an effective prognostic ERG signature for sepsis has been developed and validated.

In the current study, all these ERGs included in the risk signature have already been confirmed to be associated with endoplasmic reticulum stress. However, studies have been limited regarding their specific mechanisms of action in endoplasmic reticulum stress for sepsis. Our study mainly focused the prognostic value of ERGs, future researches are needed to clarify the specific role of these ERGs in regulating the survival of sepsis. ADRB2 (beta-2 adrenergic receptor gene) is an intron less gene located on chromosome 5q31.32. Researchers discovered that ADRB2 signaling synergizes with Toll receptors to promote rapid IL-10 release, but ADRB2 deficiency results in inflammation in bacterial infections and inflammatory colitis [26]. The enzyme 7-dehydrocholesterol reductase (DHCR7) is the ultimate enzyme in sterol biosynthesis, converting 7-dehydrocholesterol to cholesterol [27]. MAOA is a mitochondrial enzyme that catalyzes the oxidative deamination of dietary amines and monoamine neurotransmitters, such as serotonin, norepinephrine, and dopamine [28, 29]. Recent studies have shown that MAOA is correlated with sepsis-induced cardiac dysfunction [30]. Neutrophil myeloperoxidase (MPO) is a peroxidase enzyme abundant in neutrophil granulocytes. MPO level in tissue is an indicator of neutrophil migration, which reflects inflammation [31]. Neutrophils are the initial responders in sepsis, and once activated, release their granule contents, especially MPO [32]. The cellular function of PDZ domain-containing 8 protein (PDZD8) is uncertain, although There is evidence that PDZD8 is a moesin-interacting and microtubule stability regulating factor [33]. Toll-like receptor 4 (TLR4) is associated with innate immunity, as well as mediating inflammatory responses when it recognizes lipopolysaccharides (LPS) and endotoxins produced by bacteria. TLR4 is believed to have opposing effects In different conditions [34]. However, related studies of other genes in sepsis are still lacking.

Currently, there are no effective diagnostic biomarkers for sepsis [35]. Most biomarkers have been proposed as useful in the diagnosis of sepsis simply because their levels were found to be increased or decreased to a larger extent in sepsis patients than in non-sepsis patients or healthy individuals [36]. Due to these defects for the biomarkers, it is hard to applied to ICU clinical practice. In the present study, we assessed the diagnostic value of ERGs for sepsis. The results showed that most of these genes presented good diagnostic value for sepsis. However, these biomarkers require further validation and reliability studies.

To date, many studies have focused on endoplasmic reticulum stress in sepsis [37–39]. However, the biological events influenced by endoplasmic reticulum stress, especially the turbulence of immune cell infiltration, have not been well elucidated [40]. In the present study, we found that the high-risk group exhibited higher infiltration of plasma cells, M0 macrophages, resting dendritic cells, and eosinophils and lower infiltration of memory B cells, CD8 and gamma delta T cells, and M1 macrophages. This evidence for the association between ER stress and immunity highlights the importance of immunotherapy for sepsis patients with a high ERG risk score.

Despite the novel findings reported in our study and the positive implications for patients with sepsis, out study has limitations that require further investigation. First, we investigated the prognostic value of ERGs in sepsis cases, but we ignored other key genes, such as apoptosis- and oxidative stress-related genes, which might also play key roles in the development and progression of sepsis. Second, we established the risk score by the data of high-throughput sequencing, the critical mechanism of these ERGs play in sepsis should be explored intensively in the future study. Third, although the results from validation set showed our risk score had a good predictive power, the prognostic ability for patients in different countries and regions remains unclear.

## Supplementary Material

Supplementary Figures

Supplementary Table 1

Supplementary Table 2
