# Supplementary material for: Predicting the prognosis in patients with sepsis by an endoplasmic reticulum stress gene signature
Source: Aging (Albany NY). 2023 Nov 25;15(22):13434–51. doi: 10.18632/aging.205252 (PMC10713427; doi:10.18632/aging.205252)
Supplement: Supplementary Figures [file aging-15-205252-s001.pdf]

SUPPLEMENTARY FIGURES

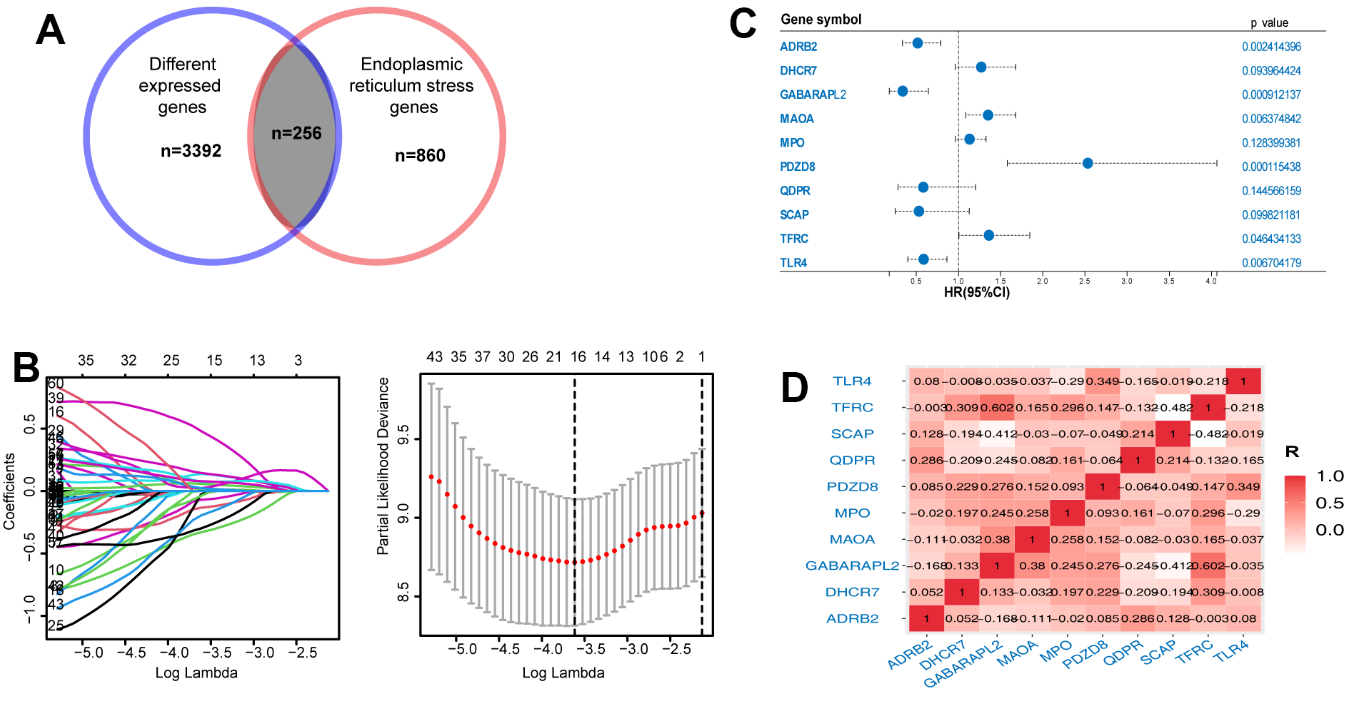

Supplementary Figure 1. Construction of signature model for risk of endoplasmic reticulum stress. (**A**) Identification of differently expressed ERGs. (**B**) LASSO coefficient of the 64 survival ERGs, and the 100-fold cross-validation for variable screening and complexity adjustment in the Lasso Cox regression model. (**C**) Construction of the ER risk signature based on the multivariate Cox regression analysis. (**D**) Heatmap represents the correlation between the identified ERGs.

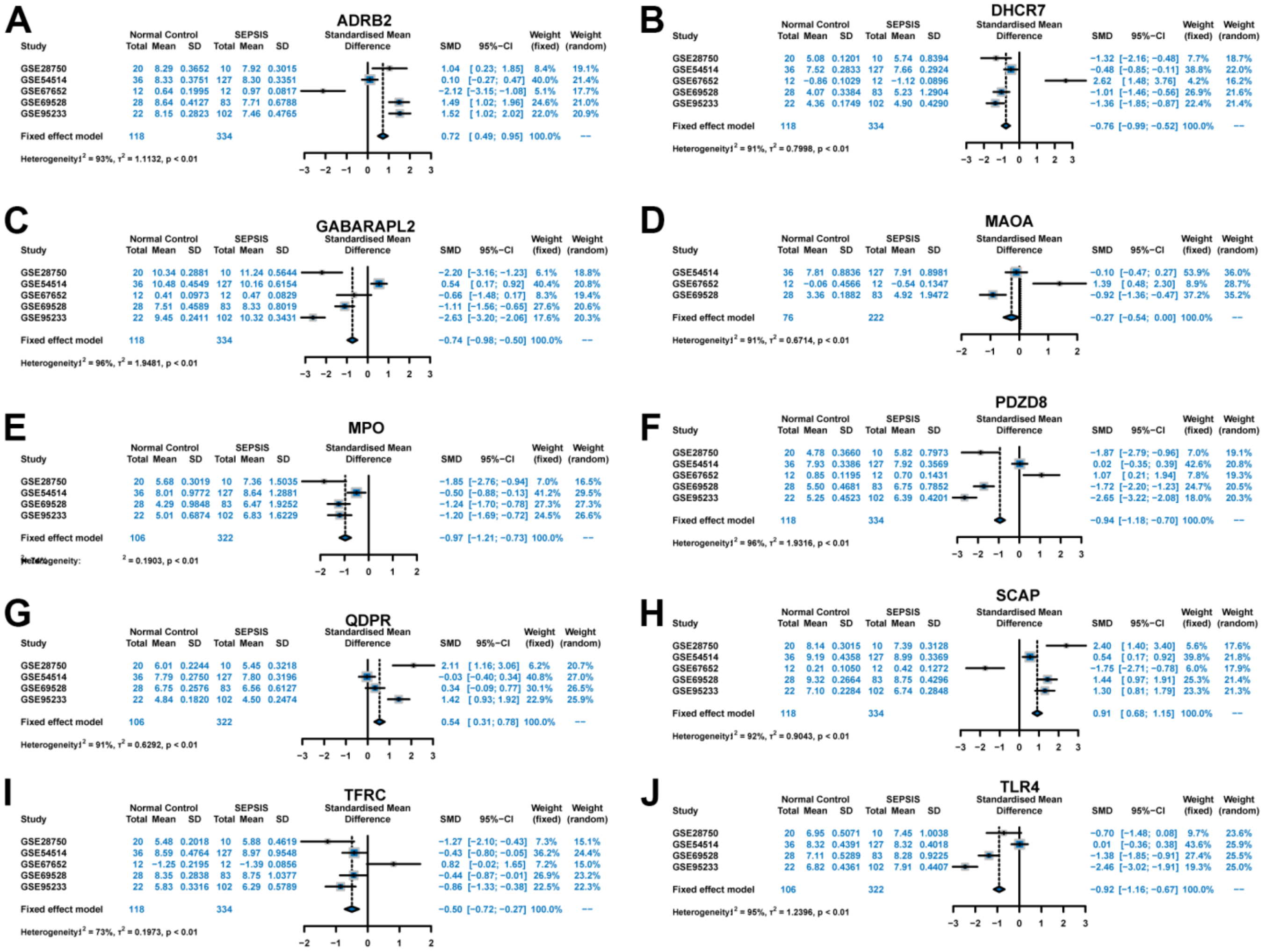

Supplementary Figure 2. Meta-analysis of the expression level (pooled results) of identified ERGs based on BIDOS database: (**A**) ADRB2, (**B**) DHCR7, (**C**) GABARAPL2, (**D**) MAOA, (**E**) MPO, (**F**) PDZD8, (**G**) QDPR, (**H**) SCAP, (**I**) TFRC, and (**J**) TLR4.

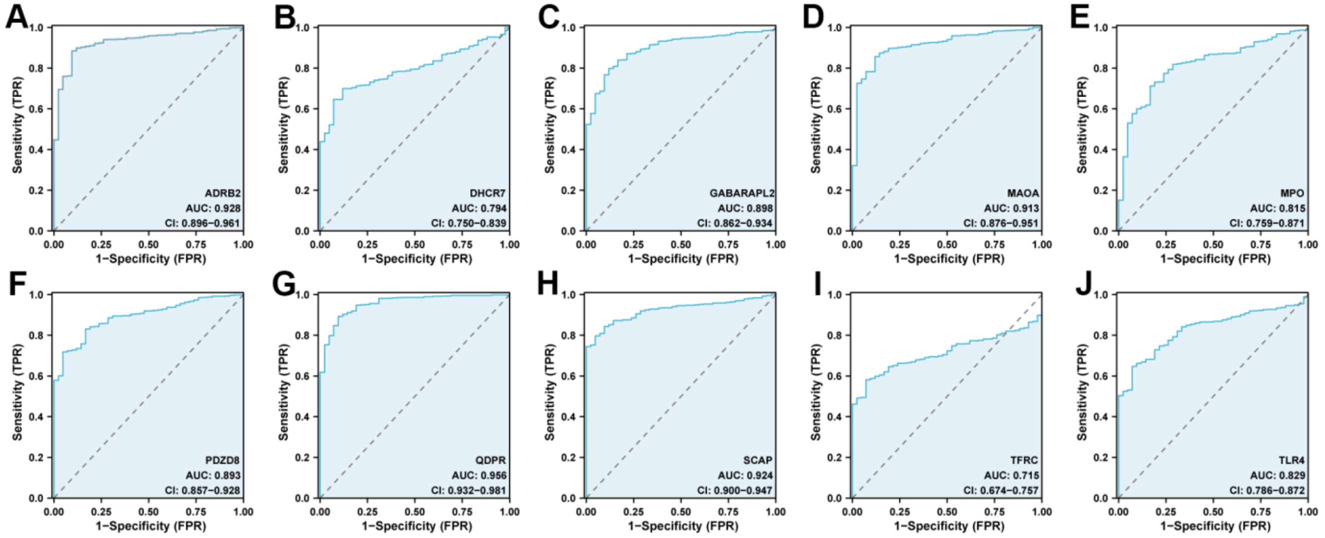

Supplementary Figure 3. Diagnostic value of identified ERGs in sepsis. ROC analyses of (**A**) ADRB2, (**B**) DHCR7, (**C**) GABARAPL2, (**D**) MAOA, (**E**) MPO, (**F**) PDZD8, (**G**) QDPR, (**H**) SCAP, (**I**) TFRC, and (**J**) TLR4.

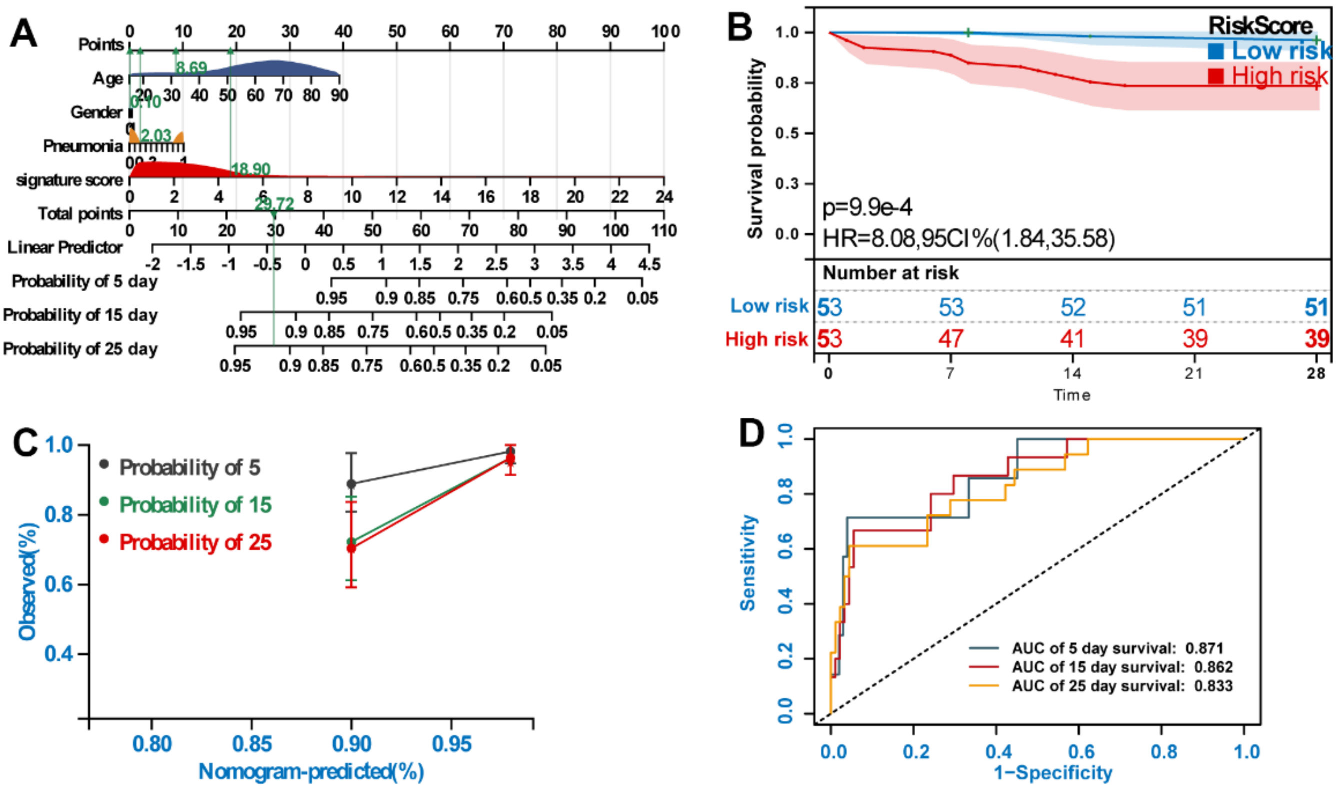

Supplementary Figure 4. Establishment of a nomogram for predicting the survival probability of patients with sepsis. (**A**) Nomogram for predicting OS developed in training dataset. (**B**) Kaplan–Meier (KM) survival curves for the survival of the nomogram. (**C**) Calibration curves of the nomogram for 5, 15, and 25 days. (**D**) Time-dependent ROC analysis of the survival-related nomogram.
