# Supplementary material for: Predicting the prognosis in patients with sepsis by an endoplasmic reticulum stress gene signature
Source: Aging (Albany NY). 2023 Nov 25;15(22):13434–51. doi: 10.18632/aging.205252 (PMC10713427; doi:10.18632/aging.205252)
Supplement: Supplementary Table 2 [file aging-15-205252-s003.pdf]

Supplementary Table 2. Selected of survival related ERGs by univariate analysis.

Gene symbol	HR	95% CI	P-value
ABCA1	0.805	0.680-0.952	0.012
ABCG1	0.697	0.520-0.934	0.016
ADRB2	0.558	0.392-0.795	0.001
AKAP9	0.503	0.256-0.991	0.047
AMFR	1.450	1.097-1.917	0.009
ANK1	1.218	1.033-1.435	0.019
ANXA2	0.578	0.406-0.823	0.002
ATF6	0.521	0.330-0.821	0.005
ATM	0.356	0.192-0.660	0.001
ATR	0.378	0.158-0.903	0.029
BCL2L1	1.262	1.012-1.573	0.039
BSG	1.506	1.124-2.020	0.006
CAPN2	0.634	0.405-0.993	0.046
CASP4	0.553	0.394-0.777	0.001
CD74	0.619	0.469-0.818	0.001
CREB1	0.556	0.328-0.941	0.029
DHCR7	1.408	1.097-1.805	0.007
EIF2AK1	1.486	1.094-2.018	0.011
ERP29	0.667	0.446-0.998	0.049
FAM120A	0.441	0.243-0.799	0.007
FKBP1B	1.477	1.179-1.849	0.001
FOXO1	0.609	0.403-0.920	0.018
FOXO3	1.405	1.076-1.835	0.012
FURIN	1.682	1.102-2.567	0.016
GABARAPL2	1.624	1.026-2.569	0.038
GRAMD1A	0.669	0.482-0.929	0.016
HERPUD2	0.605	0.407-0.898	0.013
HLA-DRB1	0.688	0.552-0.857	0.001
JAGN1	0.593	0.361-0.976	0.040
KCNJ2	0.716	0.571-0.897	0.004
LDLR	1.490	1.126-1.972	0.005
MAOA	1.408	1.174-1.689	0.000
MAPK14	0.709	0.509-0.988	0.042
MIR21	0.557	0.413-0.750	0.000
MPO	1.254	1.099-1.431	0.001
NLRP1	0.663	0.466-0.945	0.023
NLRP3	0.649	0.446-0.944	0.024
PCSK6	1.482	1.079-2.036	0.015
PDZD8	1.793	1.215-2.644	0.003
PRKCD	0.555	0.365-0.844	0.006
PTEN	0.646	0.449-0.928	0.018
QDPR	0.497	0.266-0.930	0.029
SCAP	0.380	0.203-0.712	0.003
SELP	1.301	1.035-1.636	0.024
SGK1	0.698	0.530-0.919	0.010
SLC2A1	1.697	1.348-2.135	0.000
SLC8A1	0.787	0.630-0.985	0.036
STIM2	0.395	0.193-0.808	0.011
SVIP	1.432	1.096-1.871	0.009
TAP2	0.650	0.487-0.867	0.003
TAPBPL	0.582	0.372-0.908	0.017
TARDBP	0.520	0.328-0.825	0.006
TEX2	1.843	1.274-2.666	0.001
TFRC	1.514	1.242-1.846	0.000
TGM2	1.740	1.251-2.421	0.001
THBS1	1.399	1.116-1.754	0.004
TLR4	0.637	0.473-0.859	0.003

TMEM43	0.582	0.431-0.784	0.000
TMX2	0.576	0.336-0.987	0.045
TNFRSF10B	0.729	0.549-0.967	0.028
TRAPPC5	1.818	1.020-3.241	0.043
UBXN2B	0.666	0.499-0.888	0.006
VAPA	0.483	0.290-0.804	0.005
ZDHHC2	1.451	1.019-2.065	0.039
